# Comparative Analysis of Gut Microbiota Changes in Père David's Deer Populations in Beijing Milu Park and Shishou, Hubei Province in China

**DOI:** 10.3389/fmicb.2018.01258

**Published:** 2018-06-12

**Authors:** Meishan Zhang, Minghui Shi, Mengyuan Fan, Shanghua Xu, Yimeng Li, Tianxiang Zhang, Muha Cha, Yang Liu, Xiaobing Guo, Qi Chen, Yiping Li, Shumiao Zhang, Defu Hu, Shuqiang Liu

**Affiliations:** ^1^College of Nature Conservation, Beijing Forestry University, Beijing, China; ^2^Research Department, Beijing Milu Ecological Research Center, Beijing, China

**Keywords:** Père David's deer, gut microbiota, 16s rRNA gene, high-throughput sequencing, Bacteroidetes, Firmicutes

## Abstract

This study used 16S rRNA high-throughput sequencing technology to examine the differences in gut microbiota between the Père David's deer populations in the Beijing and Shishou areas of China in order to understand the effects of *ex situ* conservation on the intestinal microflora in the Père David's deer.

**Results:** On the phylum level, the main bacteria found in the Père David's deer populations from both areas were similar: *Firmicutes* and *Bacteroidetes*. However, the relative abundances of the two groups were significantly different. Alpha diversity results indicated that there was a difference in the evenness of the microflora between the two groups, and the beta diversity results further indicated that there was a significant difference in the microflora structure between the two groups.

**Conclusions:** During the *ex situ* conservation process of the Père David's deer, their food sources may change, resulting in differences in the gut microbiota. The intestinal microflora in the Père David's deer from the same area are clustered. Therefore, the impact of changes in food on the gut microbiota of the Père David's deer should be taken into consideration during *ex situ* conservation.

## Introduction

The Père David's deer (*Elaphurus davidianus* or milu) first appeared in the early Pleistocene epoch and was a typical deer in the northern regions of China. It became extinct in China around 1900, but was bred again at the Beijing Milu Ecological Research Center in 1985 (Zhong et al., 2015). The Père David's deer is currently considered an endangered species (Bai et al., 2012). Initially, the number of Père David's deer in China was 79, but today there are more than 6,000 Père David's deer spread over 70 places throughout the country. The Père David's deer has increased significantly in number and become the representative species for the restoration and revival of endangered species in China (Zhang et al., 2012). The deer has been breeding in *ex situ* conservation areas, such as Dafeng, Jiangsu Province, Shishou, Hubei Province, Cixi Wetland, Zhejiang Province, Qianshan Deer Farm in the city of Liaoyang, and Mulan Weichang in Hebei Province (Zhang and Zhang, 2013). To better protect the re-introduced population and eventually release the captive deer, many domestic and foreign studies have focused on the feeding and management (Chen et al., 2004; Li K. et al., 2007; Meng et al., 2010), genetic breeding (Li et al., 2005a,b; Ding et al., 2009), and populations (Jiang et al., 2001; Yang et al., 2007; Wang et al., 2009) of the Père David's Deer. As their population increases, the incidence of disease has also risen. Bacteria such as *Clostridium perfringens* (Yang et al., 2004; Li C. X. et al., 2007; Zhong et al., 2007), pathogenic *Escherichia coli* (Wang et al., 1991; Zhong et al., 2007), *Clostridium septicum* (Ding, 2004), and *Pasteurella* (Yang et al., 2004) are a serious threat to the survival of the deer, and they normally infect Père David's deer in combinations. Zhang et al. (1997) analyzed the death patterns of the Père David's deer and found that the peak period of deaths was in the seasons of winter and spring. They considered digestive tract diseases to be the primary factor for the deaths of the Père David's deer in captivity. However, there are currently no detailed studies on the intestinal microorganisms in Père David's deer.

The gut microbiota is a collective term for microorganisms that live in the digestive tracts of animals, and refers to the microbial diversity formed between intestinal microorganisms and their host and environment. This diversity is interdependent, interinhibitive, and relatively stable. Unlike single-stomach animals, ruminants have a large forestomach, and the microbes are closely related to the production and health of ruminants (Malmuthuge and Le, 2017). The digestion of ruminants depends on the complex microflora in the rumen. The rumen is a feed processing plant in ruminant, and 70 to 85% of the digestible and 50% of the crude fibers in the feed are digested in the rumen. The gut microbiota is closely related to the nutritional metabolism and immune system of their host species, and an important factor in the maintenance of its health. The gut microbiome helps maintain the immune and digestive systems of the host species. Once damaged, it could lead to various diseases (Buddington and Sangild, 2011; Chinen and Rudensky, 2015; Ding et al., 2017). Most studies on the gut microbiota are based on fecal samples. Fecal microbial data reflect the overall status of microbial communities in the intestinal tract (Li C. X. et al., 2007), and are easily collectible and non-destructive. Studying the structural characteristics of the gut microbiota in Père David's deer allows us to understand their health status and provide scientific data for their *ex situ* conservation.

Studies have shown that the interaction between animals and microorganisms inside them as well as their living environment determine the state of health or disease in the animal's body (Wei, 2008). The many differences in the hydrothermal conditions and food and nutritional statuses in Père David's deer breeding areas may lead to variances in the profile of their gut microbiota, which has an impact on their health. Therefore, analyzing and comparing the differences in the gut microbiota of Père David's deer in different breeding areas will increase our understanding of changes in the composition of gut microbiota in Père David's deer populations in *ex situ* conservation sites and provide a scientific reference for us to manually improve and evaluate the health of the Père David's deer in different areas.

Therefore, this study applied 16S rRNA high-throughput sequencing to analyze Père David's deer feces in the Beijing and Shishou areas. The 16S rRNA sequencing technology combines the advantages of high-throughput sequencing and bacterial identification based on 16S rRNA genes. This facilitates the integrated study of the structures and functions of mixed bacterial strains in complex samples, rendering it possible to compare the structural differences of the gut microbiota in different regions with higher accuracy. This was conducted to enable the comparison of changes and differences of gut microbiota between the *ex situ* conservation population of semi-wild Père David's deer in Shishou, Hubei and their counterparts in Beijing Milu Park, to understand the changes in gut microbiota of Père David's deer during *ex situ* conservation, and to provide scientific guidance for such conservation. The study has great significance for the understanding of the health status of Père David's deer in *ex situ* conservation reserves, the exploration of the most suitable environment for the deer's survival, and the improvement of the success rate of *ex situ* conservation of Père David's deer.

## Materials and methods

### Basic facts of the research areas

Table below Information on Beijing Nanhaizi Père David's deer Park and Hubei Shishou Père David's deer National Nature Reserve (Liu et al., 2011).

**Table:** 

	**Beijing Nanhaizi Père David's deer Park**	**Hubei Shishou Père David's deer National Nature Reserve**
Geographic coordinates	N 39°46′ E 116°26′	N 29°49′ E 112°33′
Annual average temperature	12.6°	16.5°
Relative humidity	65%	80%
Annual average precipitation	620 mm	1,200 mm
Altitude	31.5 m	32.9–38.4 m
Area	60 hm^2^	1,567 hm^2^
Vegetation	Aquatic vegetation, shrubs, arboreal forest	Poplar forest, reed swamps, grassland, Chinese willow (*Salix matsudana*), shrubs
Population	180	550

The geographical information of the Beijing Nanhaizi Milu Park (the Beijing area) and the Hubei Shishou Milu National Nature Reserve (the Shishou area) is outlined in the respective tables above. In 1993, 1994, and 2002, 30, 34, and 30 Père David's deer, respectively, were brought to the Shishou reserve from the Beijing Milu Park. Breeding populations were established and allowed to range freely in enclosures.

The Shishou area is situated in a sub-tropical monsoon climate zone, where summers are hot and winters are cold and dry. Precipitation is relatively heavy in spring, early summer and late autumn. The eight major vegetation populations are *Populus nigra var. italica*/*Phragmites communis, Salix matsudana, Phragmites communis* / *Miscanthus floridulus, Cynodon dactylon, Leonurus artemisia, Scirpus triqueter, Scirpus yagara*, and *Eleocharis acicularis*. This study collected Père David's deer feces in the spring of 2017. In spring, the dominant species in the Père David's deer feeding areas in the Shishou area are *Phragmites communis, Roegneria kamoji, Cynodon dactylon, Hydrocotyle sibthorpioides, Leersi hexandra, Imperata cylindrical*, and *Carex argyi Levl. et Vant*.

The Beijing area is situated in a temperate and semi-humid monsoon zone, where the four seasons are distinct. Summers are hot and rainy, while winters are cold and dry. The dominant tree in the Père David's deer activity areas is *Salix matsudana*, while the dominant plant species include *Medicago sativa Linn., Eleusine indica, Eragrostis cilianensis, Digitaria sanguinalis*, and *Setaria viridis*. Due to the limitations of climate conditions, manual feeding is conducted in Beijing Milu Park in spring. The major components of the concentrated feed is 50% corn, 26% soybean meal, 11% bran, 10% barley, 2% calcium hydrogen phosphate, and 1% sea salt. Corn and barley are common feed crops that are rich in nutrients such as sugars, protein, and fat. Soybean meal is a by-product of soybean oil extraction and one of the major protein feeds for animals. Bran is a by-product obtained from the processing of wheat to flour and contains a large amount of vitamins.

### Animals and sample collection

Twelve healthy adult Père David's deer were selected from each of the Shishou and Beijing areas (Between the ages of 4 and 8, the body weight is 120–180 kg, six female elk and six male elk.), and ear tags were used to distinguish each individual deer. Sampling at the same time in mid-march 2017. Having cleaned the Père David's deer habitat the previous night, fresh fecal samples were collected in the early morning. Large, fresh, and relatively intact pieces of feces were collected. Disposable sterile gloves were worn when collecting samples to avoid human contamination. Samples were stored in sterile centrifuge tubes immediately after collection and sealed to avoid cross-contamination between samples. Immediately after sampling, all fresh fecal samples were stored in liquid nitrogen and returned to the laboratory, where they were stored at −80°C until DNA was extracted.

### DNA extraction and purification

Total bacterial DNA was extracted with the QIAamp DNA Stool Mini Kit (QIAGEN, Hilden, Germany) according to the manufacturer's protocol. The integrity of the nucleic acids were determined visually by electrophoresis on a 1.0% agarose gel containing ethidium bromide. The concentration and purity of each DNA extract were determined using a Qubit dsDNA HS Assay Kit (Life Technologies, Carlsbad, CA, United States). The extracted total DNA was preserved at −80°C.

### MetaVxTM library preparation and illumina MiSeq sequencing

Next generation sequencing library preparations and Illumina MiSeq sequencing were conducted at GENEWIZ, Inc. (Suzhou, China). DNA samples were quantified using a Qubit 2.0 Fluorometer (Invitrogen, Carlsbad, CA, United States). The 40–60 ng DNA was used to generate amplicons using a MetaVxTM Library Preparation kit (GENEWIZ, Inc., South Plainfield, NJ, United States). V3, V4 hypervariable regions of microbial 16S rDNA and ITS1 regions of fungus were selected for generating amplicons and following taxonomy analysis. GENEWIZ designed a panel of proprietary primers aimed at relatively conserved regions bordering the V3, V4, and ITS1 hypervariable regions of the bacterial and archaeal 16S rRNA gene and fungus gene. (For samples containing eukaryotic DNA, only V3 and V4 regions will be amplified). The V3 and V4 regions were amplified using forward primers containing the sequence “ACTCCTACGGGAGGCAGCA” and reverse primers containing the sequence “GGACTACHVGGGTWTCTAAT.” The ITS1 regions were amplified using forward primers containing the sequence “CTTGGTCATTTAGAGGAAGTAA” and reverse primers containing the sequence “GCTGCGTTCTTCATCGATGC.” The first round PCR, respectively, amplified the V3–V4 and ITS1 regions to obtain the target fragment and part of the adapters sequence, and the second round PCR mixed the first round PCR amplification products. At the same time, indexed adapters were added to the ends of the 16S rDNA amplicons to generate indexed libraries ready for downstream NGS sequencing on Illumina Miseq. DNA libraries were validated by Agilent 2100 Bioanalyzer (Agilent Technologies, Palo Alto, CA, United States), and quantified by Qubit 2.0 Fluorometer. DNA libraries were multiplexed and loaded on an Illumina MiSeq instrument according to manufacturer's instructions (Illumina, San Diego, CA, United States). Sequencing was performed using a 2300/250 pairedend (PE) configuration; image analysis and base calling were conducted by the MiSeq Control Software (MCS) embedded in the MiSeq instrument.

### Data analysis

The QIIME data analysis package was used for 16S rRNA data analysis. The forward and reverse reads were joined and assigned to samples based on barcode and truncated by cutting off the barcode and primer sequence. Quality filtering on joined. sequences was performed and sequence which did not fulfill the following criteria were discarded: sequence length >200 bp, no ambiguous bases, mean quality score 20. Then the sequences were compared with the reference database (RDP Gold database) using UCHIME algorithm to detect chimeric sequence, and then the chimeric sequences were removed. The effective sequences were used in the final analysis. Sequences were grouped into operational taxonomic units (OTUs) using the clustering program VSEARCH (1.9.6) against the Silva 119 database preclustered at 97% sequence identity. The Ribosomal Database Project (RDP) classifier was used to assign taxonomic category to all OTUs at confidence threshold of 0.8. The RDP classifier uses the Silva 119 database which has taxonomic categories predicted to the species level. Novel clusters (OTUs that did not match the reference database) were removed when performing analysis.

Sequences were rarefied prior to calculation of alpha and beta diversity statistics. Alpha diversity indexes were calculated using the Mothur software (Schloss et al., 2009) from rarefied samples using for richness and diversity indices of bacterial community (i.e., ACE, Chao1, Shannon, and Simpson). Principal coordinate analysis (PCoA) performed using unweighted UniFrac. A oneway analysis of similarity (ANOSIM) was performed to determine the differences among groups (Clarke and Gorley, 2006). Here, the Bray–Curtis similarity index was used as a metric of similarity between the bacterial communities based on the abundance of OTUs between samples. The heatmap figures, Venn diagrams, and ANOSIM were produced using R1, and the cladogram was generated using the online LEfSe project2. Differences in phylum and genus relative abundances are presented as means ±SD. Student's *t*-test by SPSS 25 was used for data analysis. A *P*-value < 0.05 was considered statistically significant. The raw sequences obtained in this study were available through the NCBI Sequence Read Archive (accession number SRR5839043).

## Results

### Analysis of 16S rRNA sequencing results

**Figure 1:**
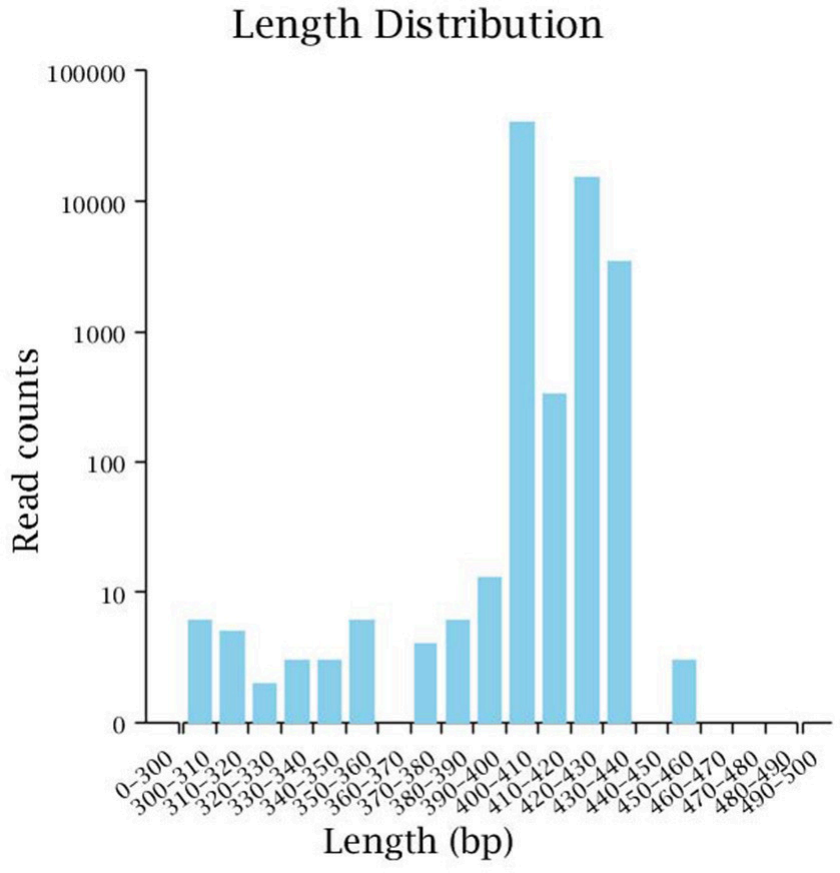
Effective sequence length distribution. The x-coordinate is the sequence length range; the y-coordinate is the reads number.

Illumina MiSeq sequencing technology was used to detect 16S rRNA gene sequences in the fecal microbiota of the semi-wild Père David's deer that were bred in the Beijing and Hubei areas. After a series of purification and filtration processes on the sequencing results, 59,837 valid sequences were obtained from each sample, and a total of 1,436,086 sequences were obtained (average length: 413.42 base pairs). The statistics of the filtered sequencing data of each sample are shown in Table 1. The reads sequences in the corresponding length range of each sample after quality control filtration were counted. The effective sequence length distribution is shown in Figure 1.

**Table 1:** Statistics of sequencing data of each sample after filtration.

**Sample**	**PE_reads**	**Effective tags**	**AvgLen(bp)**	**GC(%)**	**Effective(%)**
B1	79,947	58,904	413	52.34	73.68
B2	80,039	58,496	412	52.43	73.08
B3	79,946	57,561	412	52.33	72.0
B4	80,105	58,334	413	52.21	72.82
B5	80,378	58,855	412	52.25	73.22
B6	80,014	58,720	412	52.37	73.39
B7	80,189	59,118	412	52.34	73.72
B8	80,189	58,047	412	52.27	72.32
B9	79,714	59,990	412	52.38	75.26
B10	79,878	59,408	412	52.35	74.37
B11	79,973	58,408	414	52.1	73.03
B12	79,795	59,307	413	52.2	74.32
S1	79,714	59,021	414	52.21	73.69
S2	79,726	59,137	415	52.01	74.18
S3	80,233	59,971	414	52.04	74.75
S4	79,836	59,971	414	52.13	73.23
S5	79,946	60,503	415	51.92	75.68
S6	79,783	59,661	415	52.13	74.78
S7	80,022	59,005	414	52.14	73.74
S8	79,961	60,124	414	52.25	75.19
S9	79,773	59,581	414	51.97	74.69
S10	79,896	59,695	414	52.15	74.72
S11	80,309	60,391	416	51.83	75.2
S12	80,250	60,440	414	52.11	75.31

*Sample, name of sequencing sample; PE reads, paired-end reads number obtained by sequencing; Raw tags, number of original sequences obtained by splicing paired-ended reads; Clean tags, number of optimized sequences after filtering the original sequence; Effective tags, Clean tags Number of effective sequences after chimera filtered by clean tags; AvgLen (bp), Average sequencing length of the sample; GC (%), GC content of the sample, i.e., the percentage of G and C bases relative to the total number of bases; Effective (%): Proportion of effective tags among PE reads*.

**Figure 2:**
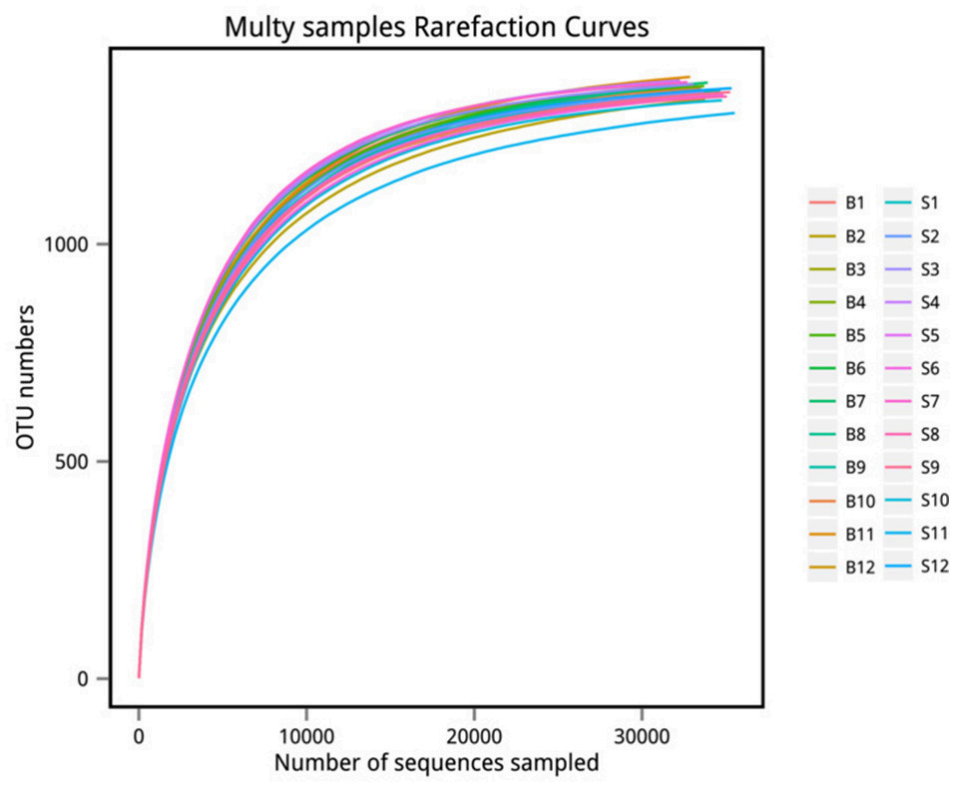
Rarefaction curve. The x-coordinate is the number of sequences sampled and the y-coordinate is the number of observed OTUs. Each curve in the graph represents a sample, which is labeled with a different color. The number of OTUs increases with the sequencing depth. When the curve becomes stable, the number of detected OTUs does not increase with the expansion of extracted data, indicating a time when the amount of sequencing data is reasonable.

The number of sequences in each sample's OTU was obtained within the 97% sequence similarity threshold. By comparing the OTU representative sequences with a microbial reference database, we obtained classification information for each species corresponding to each OTU. The bacteria that could be detected were classified into 12 phyla, 22 classes, 27 orders, 47 families, and 94 genera. At each level (phylum, class, order, family, genus and species) the composition of each sample community was calculated. The number of each species at different levels are shown in Table 2, and the total number of OTUs covered by each sample in their subordinate levels are shown in Table 3. The dilution curves of the OTUs measured in this study indicated that the number of OTUs increased with the depth of sequencing. The final curve became stable, signifying that the amount of sequencing data is somewhat reasonable (Figure 2).

**Table 2:** Statistics of OTU species of samples on various levels.

**Sample**	**Kindom**	**Phylum**	**Class**	**Order**	**Family**	**Genus**	**Species**
B1	1	12	23	28	47	91	6
B2	1	12	21	26	44	87	5
B3	1	12	22	26	43	88	6
B4	1	12	21	25	43	89	6
B5	1	12	20	25	42	87	6
B6	1	12	20	25	43	88	6
B7	1	12	21	24	44	88	5
B8	1	12	20	24	44	88	6
B9	1	12	20	23	40	86	6
B10	1	12	22	26	45	90	6
B11	1	12	23	26	44	89	6
B12	1	12	22	25	43	88	6
S1	1	11	21	24	44	90	6
S2	1	12	23	25	43	91	6
S3	1	12	23	26	45	91	6
S4	1	12	23	25	45	90	5
S5	1	12	23	25	43	90	6
S6	1	11	21	23	42	89	6
S7	1	12	23	25	45	91	6
S8	1	11	19	21	42	90	6
S9	1	11	20	22	42	89	6
S10	1	11	21	23	42	88	6
S11	1	11	17	21	39	84	4
S12	1	12	22	23	43	91	6

*B represents Père David's deer samples from Beijing Nanhaizi Milu Park and S represents Père David's deer samples from Shishou Milu Nature Reserve, Hubei Province*.

**Table 3:** Statistics of OTU clustering results of samples on various levels.

**Sample**	**Kindom**	**Phylum**	**Class**	**Order**	**Family**	**Genus**	**Species**
B1	33,145	33,104	33,104	33,103	32,962	32,169	31,746
B2	33,761	33,727	33,727	33,727	33,496	32,286	31,746
B3	32,277	32,163	32,163	32,161	32,054	31,280	30,894
B4	32,879	32,862	32,862	32,862	32,727	31,969	31,354
B5	33,708	33,691	33,691	33,691	33,546	32,572	32,097
B6	32,741	32,704	32,704	32,704	32,584	31,462	31,037
B7	33,915	33,907	33,907	33,905	33,756	32,711	31,928
B8	33,354	33,348	33,348	33,348	33,206	32,218	31,853
B9	34,619	34,616	34,616	34,616	34,468	33,518	32,688
B10	33,525	33,411	33,411	33,411	33,293	32,430	32,059
B11	32,866	32,790	32,790	32,789	32,678	31,715	31,112
B12	31,720	31,547	31,547	31,546	31,430	30,741	30,386
S1	33,535	33,532	33,532	33,525	33,344	32,208	31,015
S2	33,684	33,672	33,672	33,429	33,339	32,472	31,495
S3	33,079	33,040	33,040	33,036	32,922	31,967	31,241
S4	32,219	32,208	32,208	32,207	32,072	31,337	30,544
S5	34,816	34,791	34,791	34,776	34,656	33,925	32,927
S6	34,625	34,624	34,624	34,229	34,096	32,891	31,626
S7	32,699	32,683	32,683	32,680	32,544	31,822	31,039
S8	35,024	35,018	35,018	35,002	34,858	33,822	32,740
S9	35,245	35,234	35,234	35,202	35,089	33,950	33,077
S10	34,763	34,761	34,761	34,364	34,228	32,999	31,741
S11	35,525	35,520	35,520	35,520	35,458	33,917	33,592
S12	35,345	35,341	35,341	35,071	34,981	34,142	33,175

### Comparison of core intestinal microflora between Père David's Deer in the Beijing and Shishou areas

**Figure 3:**
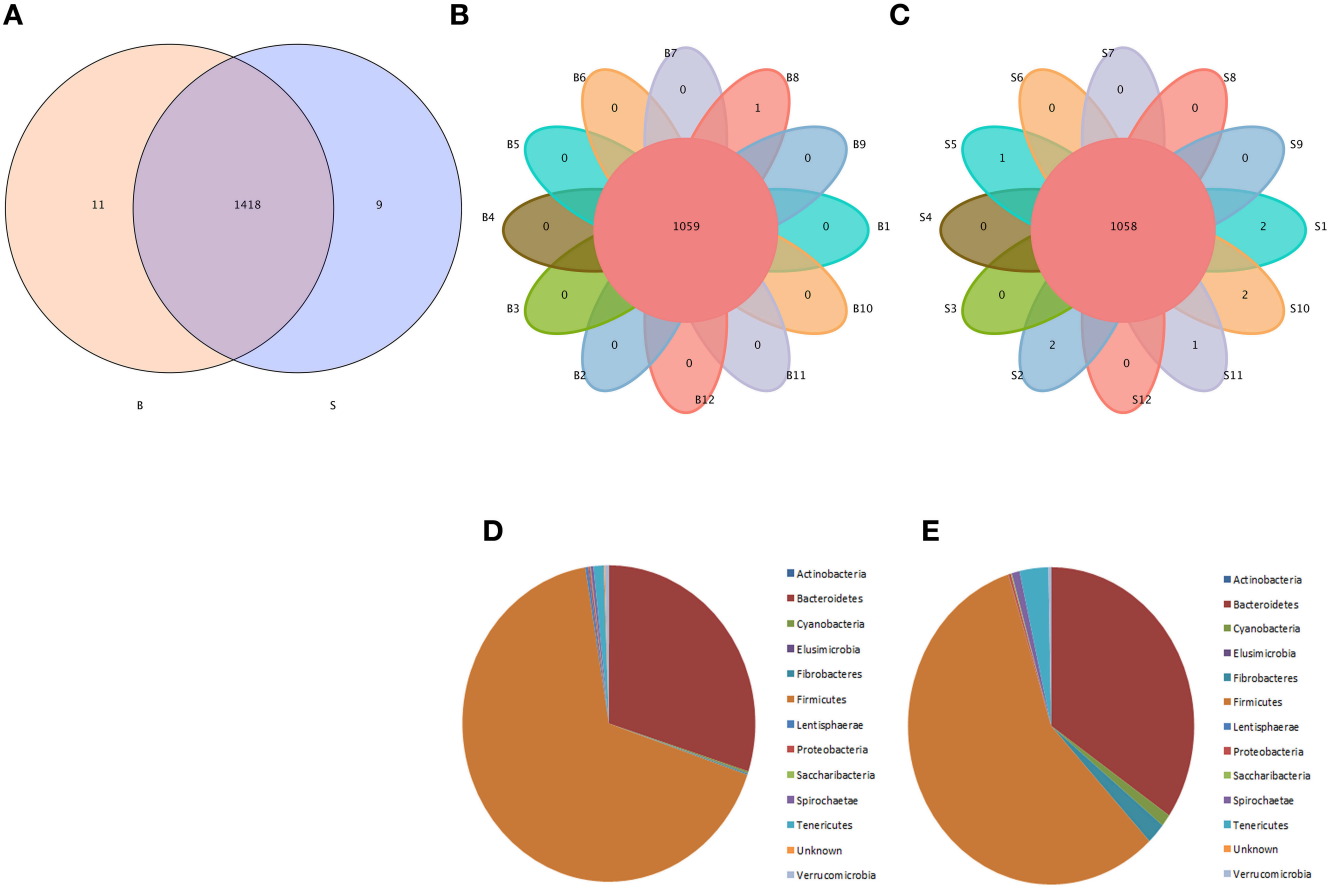
Venn diagram and pie charts. The Venn diagrams show the numbers of OTUs (97% sequence identity) that were shared or not shared by B and S individuals, respectively, depending of overlaps. For this presentation, two individuals had to be combined (e.g., C1_2) thereby reflecting the number of OTUs shared by both individuals. **(A)** The number of OTUs shared by B and S. **(B)** The number of OTUs shared by B. **(C)** The number of OTUs shared by S. The pie diagram shows the 20 most abundant taxa (calculated over the combined dataset) in B and S. **(D)** B, **(E)** S.

Venn diagrams were used to confirm the core intestinal microflora of the Père David's deer in the Beijing and Shishou areas. The bacterial populations common to all individuals in each group were considered the core microflora. As shown in Figure 3, the number of OTUs shared by all individual Père David's deer in the Beijing and Shishou areas was 1,438 (Figure 3A), while the number of OTUs was 1,059 for individual deer in the Beijing area (Figure 3B) and 1,058 for individual deer in the Shishou area (Figure 3C). The main bacterial phyla in the intestines of each group of Père David's deer are shown in Figure 3D (the Beijing area) and Figure 3E (the Shishou area). The two dominant phyla in these sequences were *Firmicutes* and *Bacteroidetes*.

**Figure 4:**
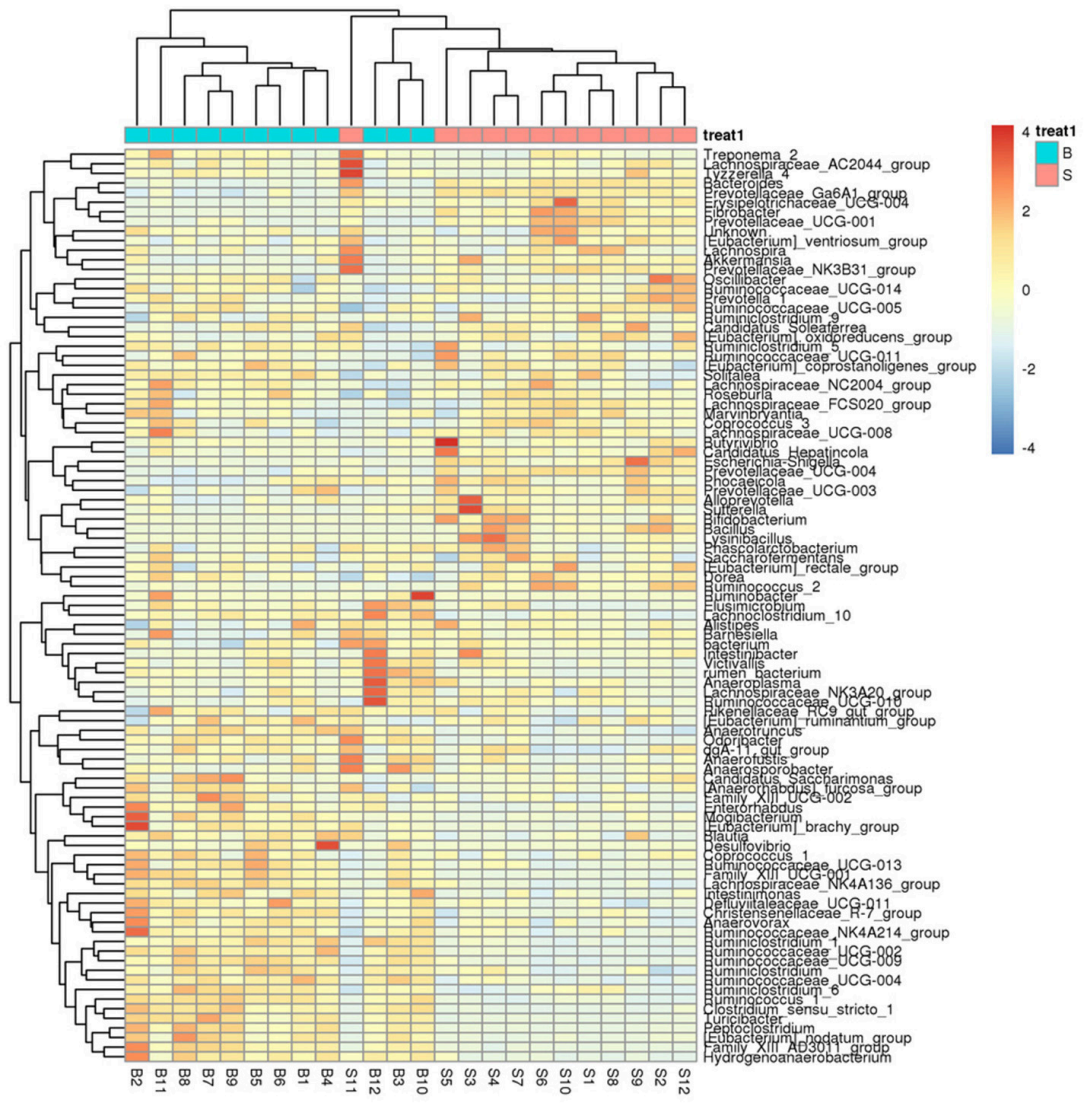
Heatmap showing richness of species at each level. The corresponding values of the heatmap are the *Z* values obtained by normalizing the relative abundance of species on each row. The color gradient from blue to red indicates a low to high relative abundance. The vertical clustering indicates the similarity in the richness of different species among different samples. The closer the distance between two species, the shorter the branch length, indicating greater similarity in richness between the two species. Horizontal clustering indicates the similarity of species richness in different samples. Similarly, the closer the distance between two samples, the shorter the branch length, indicating greater similarity in richness of species between the two samples.

A heatmap (Figure 4) is a graphical representation that uses a system of colored gradients to represent the size of values in a data matrix and cluster data based on species or the abundance similarity of samples. High-abundance and low-abundance species are clustered by color gradient and similarity to reflect the similarities and differences between multiple sample communities. A heatmap analysis was performed based on the species composition and relative abundance of each sample to extract the species at each taxonomic level. Mapping was achieved using R language tools, and a heatmap cluster analysis was performed at each of the phylum, class, order, family, genus, and species level.

### Diversity analysis of microbial communities in Père David's Deer in the Beijing and Shishou areas

#### Alpha diversity analysis

Alpha diversity reflects the richness and diversity of a single sample species and has several indices for measurement, such as the Chao1, ACE, Shannon, and Simpson indices. The Chao1 and ACE indices measure the richness of species (i.e., the number of species), whereas the Shannon and Simpson indices measure the diversity of species and are affected by the richness and community evenness of the sample community. In the case of equal richness, a higher community evenness among the species in the community is considered greater diversity, and the higher the Shannon index, the lower the Simpson index, which indicates higher diversity among the species in the sample (Wang and Wang, 2011). The completeness of the sequencing was tested by Good's coverage, which was close to 99% in this study, indicating that the majority of the bacterial species present in the sample had been detected.

**Figure 5:**
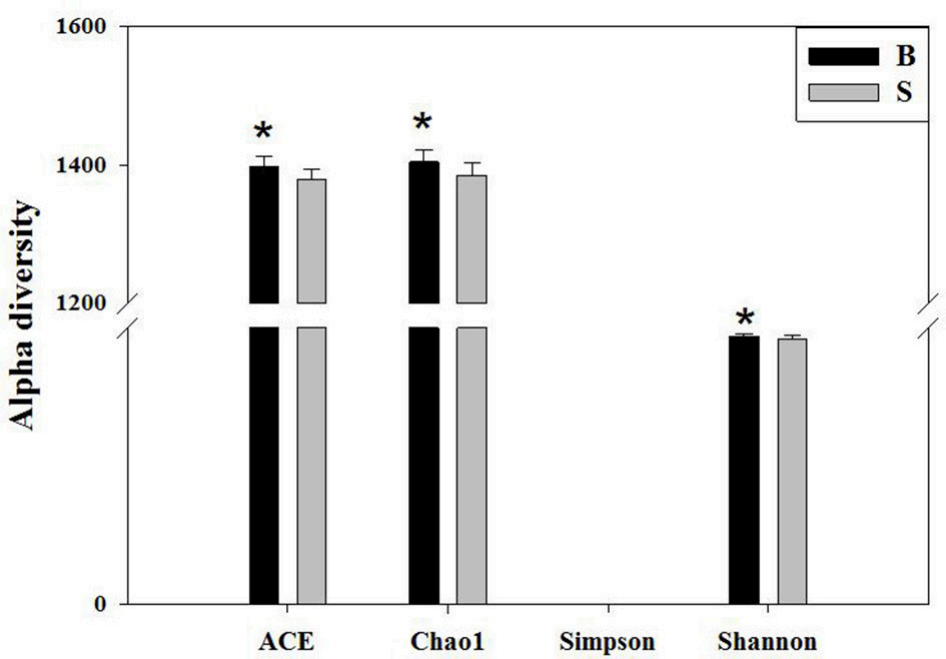
Comparison of alpha diversity indices of gut microbiota in the Beijing and Shishou areas. ACE: an index used to estimate the number of OTUs in a community. It is one of the most commonly used indices for estimating species in ecology. Chao: an index that uses the Chao1 algorithm to estimate the number of OTUs included in a sample. Chao is commonly used in ecology to assess the total number of species. Shannon: an index often used to reflect alpha diversity and estimate microbial diversity in a sample. Simpson: a diversity index commonly used in ecology to quantitatively describe the biodiversity of a geographical area. ^*^P < 0.05.

We calculated the alpha diversity (ACE, Chao1, Shannon, Simpson, and Good's Coverage) for the gut microbiota in Père David's deer in the Beijing and Shishou areas, as shown in Figure 5. There was a significant difference in the ACE, Chao1, and Shannon indices between the Beijing and Shishou areas (*P* < 0.05), but no significant difference was found in the Simpson index of the two areas (*P* > 0.05). Therefore, the alpha diversity indices of the two areas are considered to be significantly different (*P* < 0.05).

#### Beta diversity analysis

**Figure 6:**
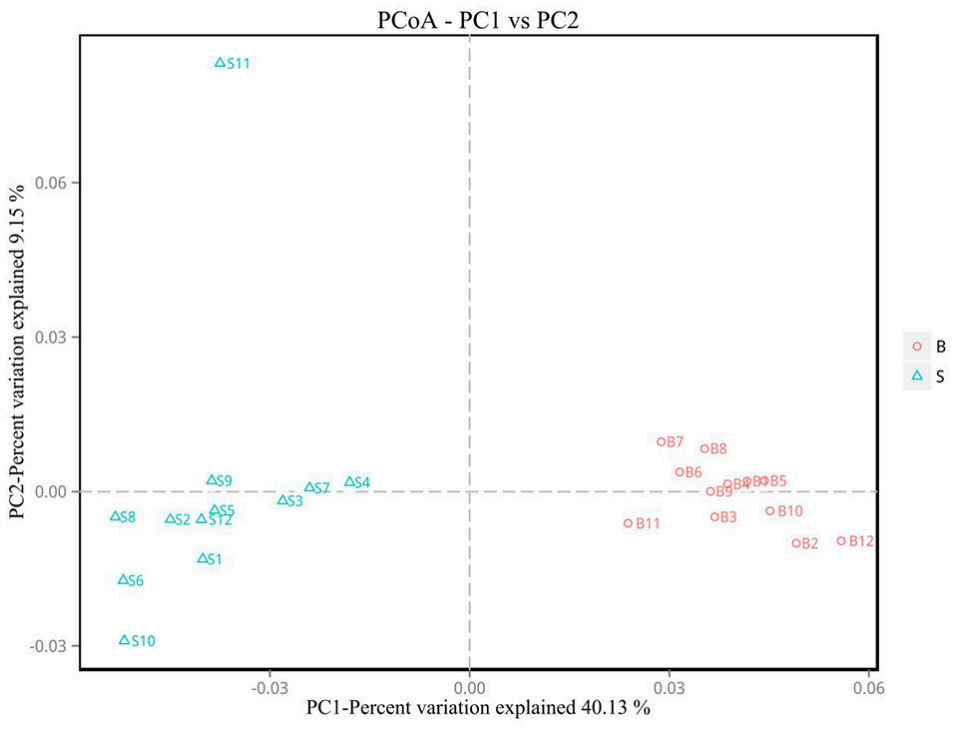
PCoA plot. Principal coordinate analysis (PCoA) plot. Red dots represent Père David's deer samples from the Beijing area, and blue squares represent Père David's deer samples from the Shishou area. Samples in the same group are represented by the same color and shape. PC1 vs. PC2 is the PCoA plot obtained from the first and second main coordinates; the x-axis and y-axis represent the first and second main coordinates, respectively. The percentage of the main coordinates represent the relative contribution of this coordinate to sample differences, which is a measure of the amount of original information extracted by this main coordinate. The distances between the sample points represent the similarity of microbiota in the samples. A closer distance represents higher similarity and samples that cluster together are composed of similar microbiota.

Principal Coordinate Analysis (PCoA) (Li et al., 2005b) is an approach to sequencing using dimension reduction. It assumes that where there is data to measure the differences or distances between N number of samples, this method can be used to plot a rectangular coordinate system representing the N samples as N points, and the square of the Euclidean distance between the points is exactly equal to the original differential data. Thus, qualitative data is converted into quantitative data and the most important elements and structures are extracted from the multi-dimensional data. The classification of multiple samples can be achieved through PCoA, which further demonstrates the differences in diversity between samples. As observed in PC1 vs. PC2 shown in Figure 6, samples that are closer together in the graph indicate greater similarity.

### Analysis of the differences in gut microbiota between Père David's Deer in the Beijing and Shishou areas at the phylum and genus levels

**Figure 7:**
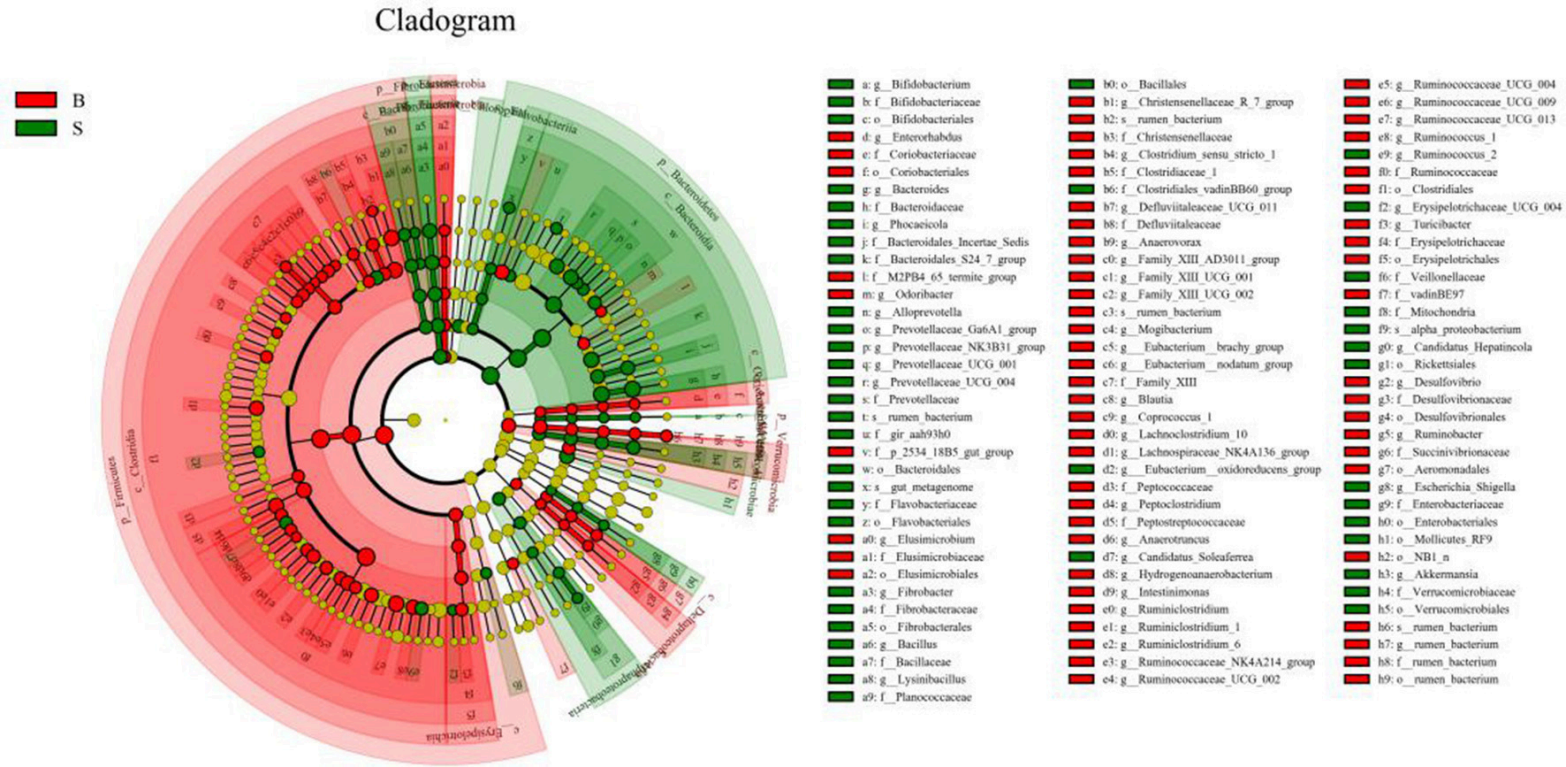
LEfSe analysis of evolutionary tree. The circles from the inside to the outside of the evolutionary tree represent the classification from the phylum to the species level. Each small circle on a different classification level represents a classification below that level, and the diameter of the small circle is proportional to the relative abundance. Species with no significant differences are all represented by the color yellow, whereas the other significant different species are colored according to the group with the highest abundance to which the species belong. Different colors indicate different groups, and the nodes of different colors indicate the microorganisms that play an important role in the group represented by the color.

**Figure 8:**
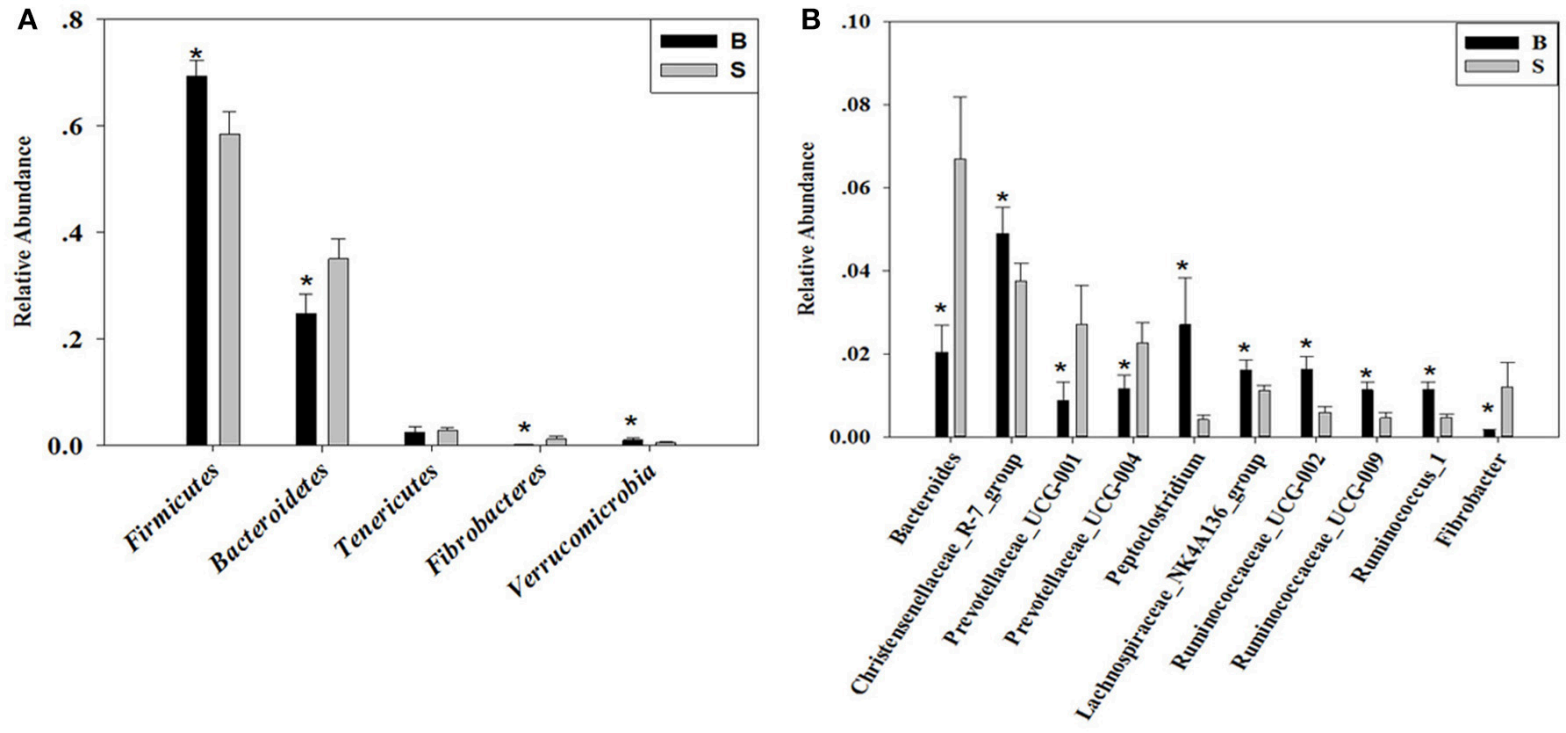
Differences in relative abundance (mean % SD) of 5 major bacterial phyla and 10 major bacterial genus between the B and S groups. **(A)** Relative abundance (mean % SD) of five major bacterial phyla between the B and S groups. **(B)** Relative abundance (mean % SD) of 10 major bacterial genus between the B and S groups. The significance of Firmicutes, Bacteroidetes, Fibrobacteres, and Verrucomicrobia was determined using the independent-sample t-test, whereas the non-parametric Mann–Whitney U test was used to examine the significance of Proteobacteria. ^*^represents *P* < 0.05.

The cladogram (Figure 7) showed differences in 88 taxa between B and S. And Figure 8 shows the differences in relative abundances at the phylum level of the top 5 bacterial communities and genus level of the top 10 bacterial communities in the Père David's deer samples from the Beijing and Shishou areas. In the Père David's deer from the Beijing area, the relative abundance of Firmicutes and Verrucomicrobia were significantly higher than that in the Père David's deer from the Shishou area (*P* < 0.05). In contrast, the relative abundances of the Bacteroidetes and Fibrobacteres phyla in the Shishou area were significantly lower than in the Beijing area (*P* < 0.05). Both groups did not show any significant differences in the relative abundances of Tenericutes (*P* > 0.05). At the genus level, the relative abundances of Christensenellaceae_R-7_group, Peptoclostridium, Lachnospiraceae_NK4A136_group, Ruminococcaceae_UCG-002, Ruminococcaceae_UCG-009 and Ruminococcus_1 in the Beijing area were significantly higher compared with the Shishou area (*P* < 0.05), while the relative abundance of Bacteroides, Prevotellaceae_UCG-001, Prevotellaceae_UCG-004, and Fibrobacter was lower in the Beijing area than in the Shishou area (*P* < 0.05).

## Discussion

As research into the mammalian gut microbiota has deepened and high-throughput sequencing technology extensively applied, we have seen progress in the study of the relationship between the gastrointestinal micro-ecology and health of wildlife animals. The study of mammalian gut microbiota has currently gone deep into the gene level and has even begun to explore their mechanisms of action. Relevant research has extended from simple analyses on microbiota composition to function analyses, which involve multiple aspects, such as feeding patterns, nutrition, and habitats (Ding et al., 2017). As an important and rare wild animal in China, the Père David's deer plays an essential role in demonstrating how other endangered species can be protected through the *ex situ* conservation of some existing deer populations as well as the application of artificial management and reproduction to expand their populations. Since the Shishou Nature Reserve in Hubei Province re-introduced the Père David's deer from Beijing Milu Park in 1993, the population has grown to 550, three times the deer population of Beijing. However, due to technical limitations, there is relatively limited comparative analysis on the health status between the deer populations in the two areas, especially the analysis of the differences in their gut microbiota. This study applied 16S rRNA Illumina MiSeq high-throughput sequencing technology for the first time to compare the core gut microbiota between the two Père David's deer populations, analyzed the diversity of microbial communities, and conducted a differential analysis at the phylum and genus levels. Such analyses have expanded our understanding of the health status of deer populations in *ex situ* conservation sites and provided a scientific basis for monitoring the health status of the Père David's deer.

The analysis results indicated that the amount of sequencing data in this study was reasonable (Figures 1, 2). As seen in Figure 3, the gut microbiota in the Père David's deer populations in the Beijing and Shishou areas have a high degree of similarity (95.66%), with the common phyla being *Firmicutes* and *Bacteroidetes*. This finding was consistent with the majority of studies on the gut microbiota of ruminant animals (Sundset et al., 2007; Guan et al., 2017; Li et al., 2017). In terms of alpha diversity, the Chao1, ACE, and Shannon indices of the gut microbiota were significantly different between the Père David's deer populations of the Beijing and Shishou areas (*p* < 0.05) (Figure 5). Regarding beta diversity, the gut microbiota of both deer populations displayed two clear clusters (Figure 6). Thus, the richness and evenness of the intestinal microorganisms of the Père David's deer populations are still dissimilar. We observed from the analysis in Figure 6 that there were greater differences between the deer populations in the Beijing and Shishou areas, while the differences in microbial communities within the same area were smaller.

The gut microbiome consisting of the host and the gut microbiota maintains the host's immune and digestive systems. The biodiversity and richness play an important role in maintaining the host's normal physiological functions, but is also affected by the host (Koboziev et al., 2014). The composition and functions of the gut microbiota is closely related to the health status of the host, and dysbiosis is the cause of many diseases. On the surface, dietary patterns are one of the key factors affecting the gut microbiota (Zheng et al., 2014), but differences between the gut microbiota in different geographical regions may also be due to other factors, such as host genotype, age, disease, probiotics, and drugs, all of which affect the structure and functions of the gut microbiota (Kovács et al., 1972). As the Père David's deer population in the Shishou Nature Reserve was originally transferred from the Beijing Milu Park and developed during the last century, the genotypes of individual deer from the two areas were not significantly different. In this study, we selected healthy adult Père David's deer of similar age and size as test subjects at approximately the same time, and the differences between the research areas and the samples were mainly the vegetation types caused by climatic conditions, which led to differences in the food sources of the deer populations. In spring, the deer in the Beijing area are manually fed, while in the Shishou area the feed on natural food sources. Therefore, by analyzing the differences in the food sources of the two areas could explain the biodiversity of the gut microbiota.

At the phylum level, the richness of *Firmicutes* in the intestines of the Père David's deer in the Beijing area was significantly higher than that in the Shishou area, whereas for *Bacteroidetes* it was exactly the opposite (Figure 8A). The main function of the *Firmicutes* (major food analysis) in the intestines is to hydrolyze carbohydrates and proteins, while *Bacteroidetes* (major food analysis) is responsible for the metabolism of steroids, polysaccharides, and bile acids, helping the host in the absorption of polysaccharides and the synthesis of proteins (Xu et al., 2003; Bäckhed et al., 2005). Other studies have found that a higher *Bacteroidetes* and *Firmicutes* content promotes animal fat deposition; this effect was more evident for *Firmicutes* than *Bacteroidetes* (Bäckhed et al., 2004; Turnbaugh et al., 2006). Moreover, it was found that the addition of soybean isoflavone aglycon in the diets of ruminantia, such as yellow cattle, enhances energy acquisition and stimulates the production of fat (Zhou, 2016). Hence we may infer that the significant difference in the amount of *Firmicutes* in the intestines of deer from the Beijing and Shishou areas may be because the deer diet in the Beijing area contains a certain proportion of soybean meal in springtime.

The results (Figures 4, 7, 8B) indicated that the relative abundance of *Bacteroides, Prevotellaceae*, and *Fibrobacter* of the Père David's deer in the Beijing area was significantly lower than those in the Shishou area on the family and genus levels (*p* < 0.05). The relative abundance of *Christensenellaceae, Peptoclostridium, Lachnospiraceae, Ruminococcaceae*, and *Ruminococcus* was the opposite. *Bacteroides* was also found to have other functions, such as promoting the improvement of the host's immune system and maintaining the balance of the gut microbiome (Hooper et al., 2001; Hooper, 2004; Sears, 2005; Jiang et al., 2014) studied changes in certain bacteria in the intestinal tracts of patients with ulcerative colitis, they found an increased number of *Bacteroides*, which has an inflammatory effect on patients with ulcerative colitis. Studies have also found that after adding soybean isoflavone aglycone to the diet of growing Jinjiang cattle, the richness of Bacteroides in the Bacteroidaceae family reduces, indicating that the aglycon lessens the inflammatory response in animals (Zhou, 2016). This study yielded similar results, as the abundance of *Bacteroidetes* and *Bacteroides* was significantly higher in the gut microbiota of Père David's deer in the Shishou area, compared to those in the Beijing area. The reason may be that the addition of soybean meal to the deer food in Beijing reduces the intestinal inflammation in the deer, whereas in the Shishou area the deer lack plants that contain soybean aglycon. Thus, more *Bacteroidetes* bacteria are needed to regulate the intestinal environment of the deer.

The experimental results indicated that the main types of intestinal microflora in Père David's deer are similar to those of humans (*Firmicutes* and *Bacteroidetes*) (Arumugam et al., 2011), due to the difference in food, People from different regions had significantly different in intestinal microbial communities. Therefore, dietary structure may have a significant impact on the microbial community in the intestines of deer. This finding has been verified in similar studies on other ruminants (Pitta et al., 2014). Moreover, a considerable number of studies have shown that differences in diet structure not only have an impact on the changes in the host's gut microbiota, but may also cause other physiological reactions or even diseases in animals. Studies have shown that different bacterial communities have different effects on the digestion of carbohydrates, protein, and cellulose in ruminant diets, not only affecting the host's nutrient utilization, but also possibly causing gastrointestinal diseases and obesity in the host (Bäckhed et al., 2005; Han et al., 2015). Hence, changes in the structure of the gut microbiota are mainly affected by dietary conditions, and the differences in feed composition are an inducing factor. For example, our results suggest that the differences in feed composition between the Beijing and Shishou areas explain the differences in the intestinal microorganisms of the Père David's deer. Nevertheless, whether the specific functions of different bacteria have an impact on the host's digestion and absorption and whether the change of gut microbiota after migration leads to diseases in the host and affects the health status of the deer population are issues yet to be investigated in future research.

In conclusion, our results showed that the gut microbiota of the Père David's deer population in the Shishou area is different from that of the Beijing area. This may be due to the fact that the deer population in Shishou has moved from Beijing to a new environment and has adapted to changes in their food and environment, leading to changes in their gut microbiota. Some researchers have analyzed the composition of the gut microbiota of tens of mammals and found that the diversity of the host's gut microbiota varies according to feeding patterns, gradually increasing from carnivorous, omnivorous, to herbivorous animals. Due to the differences in the digestive tract's anatomical structure and physiological metabolism, there is also a wide variation in the structures and functions of the gut microbiota (Ley et al., 2008). Therefore, after the Père David's deer migrates to a new environment, the composition and diversity of the gut microbiota are directly affected by changes in the natural environment and deer's feed, thus may impacting the adaptive changes of the digestive and immune health of the deer. To re-introduce the Père David's deer, we must ensure that there is reasonable feeding, planting of edible plants necessary for the deer's health, and scientific supervision over the deer. This study analyzed the gut microbiota of the Père David's deer in different geogrpahical areas and offered insights into the health status of the gut of the deer in China during the *ex situ* conservation process, providing an empirical reference and auxiliary measures for the scientific feeding and management of the Père David's deer.

## Author contributions

MZ, MS, SZ, DH, SL conceived and designed the study. MZ, SX, YmL, TZ and MC performed the experiments. YL, XG, QC, and YpL processed the date. MZ, MS and MF wrote the paper. MF and MS reviewed and edited the manuscript. All authors read and approved the manuscripts.

### Conflict of interest statement

The authors declare that the research was conducted in the absence of any commercial or financial relationships that could be construed as a potential conflict of interest.

## References

[B1] Arumugam M. Raes J. Pelletier E. (2011). Enterotypes of the human gut microbiome. Nature 474:666 10.1038/nature09944 PMC3728647 21508958

[B2] Bäckhed F. Ding H. Wang T. Hooper L. V. Koh G. Y. Nagy A.. (2004). The gut microbiota as an environmental factor that regulates fat storage. Proc. Natl. Acad. Sci. U.S.A. 101, 15718–15723. 10.1073/pnas.0407076101 15505215 PMC524219

[B3] Bäckhed F. Ley R. E. Sonnenburg J. L. Peterson D. A. Gordon J. I. (2005). Host-bacterial mutualism in the human intestine. Science 307, 1915–1920. 10.1126/science.1104816 15790844

[B4] Bai J. D. Zhang L. Y. Zhong Z. Y. Dong J. (2012). Research progress on the development status of Chinese *Elaphurus davidianus* population. China Anim. Husband. Veterin. Med. 39, 225–230.

[B5] Buddington R. K. Sangild P. T. (2011). Companion animals symposium: development of the mammalian gastrointestinal tract, the resident microbiota, and the role of diet in early life. J. Anim. Sci. 89, 1506–1519. 10.2527/jas.2010-3705 21239667

[B6] Chen Y. Y. Bao L. Y. Sai D. J. Wang J. L. (2004). The improvement of Habitat in Dafeng's deer reserve. J. Shandong Norm. Univ. 9, 74–76.

[B7] Chinen T. Rudensky A. Y. (2015). The effects of commensal microbiota on immune cell subsets and inflammatory responses. Immunol. Rev. 245, 45–55. 10.1111/j.1600-065X.2011.01083.x 22168413

[B8] Clarke K. R. Gorley R. N. (2006). PRIMER v6: User Manual/Tutorial. Plymouth: Plymouth Marine Laboratory.

[B9] Ding Y. H. (2004). Study on Père David's Deer in China. Changchun: Jilin science and Technology Press.

[B10] Ding Y. H. Ren Y. J. Xu A. H. Xie S. B. Hou L. B. (2009). Behaviors difference of Père David ' s deer harem master between the wild deer and the captive deer during rut. J. Nanjing Norm. Univ. 32, 114–118.

[B11] Ding Y. Wu Q. Hu Y. B. Wang X. Nie Y. G. Wu X. P. (2017). Advances and prospects of gut microbiome in wild mammals. Acta Theriol. Sin. 37, 399–406.

[B12] Guan Y. Yang H. T. Han S. Y. Feng L. Wang T. Ge J.. (2017). Comparison of the gut microbiota composition between wild and captive sika deer (*Cervus nippon hortulorum*) from feces by high-throughput sequencing. AMB Expr. 7, 212–225. 10.1186/s13568-017-0517-8 29170893 PMC5700909

[B13] Han X. Yang Y. Yan H. Wang X. Qu L. Chen Y. (2015). Rumen bacterial diversity of 80 to 110-day-old goats using 16S rRNA sequencing. PLoS ONE 2:e0117811 10.1371/journal.pone.0117811 PMC4336330 25700157

[B14] Hooper L. V. (2004). Bacterial contributions to mammalian gut development. Trends Microbiol. 12, 129–134. 10.1016/j.tim.2004.01.001 15001189

[B15] Hooper L. V. Wong M. H. Thelin A. Hansson L. Falk P. G. Gordon J. I.. (2001). Molecular analysis of commensal host-microbial relationships in the intestine. Science 291, 881–885. 10.1126/science.291.5505.881 11157169

[B16] Jiang M. Gao H. L. Yao P. (2014). Quantification of intestinal microflora in ulcerative colitis patients using real-time PCR. World Chin. Digest. Mag. Clin. Pract. 22, 596–600. 10.1155/2016/9186232

[B17] Jiang Z. G. Zhang L. Y. Yang R. S. Xia J. S. Rao C. G. Yu H. (2001). Density dependent growth and population management strategy for Père David's deer in China. Acta Zool. Sin. 47, 53–58.

[B18] Koboziev I. Webb C. R. Furr K. L. Grisham M. B. (2014). Role of the enteric microbiota in intestinal homeostasis and inflammation. Free Radic. Biol. Med. 68, 122–133. 10.1016/j.freeradbiomed.2013.11.008 24275541 PMC3943931

[B19] Kovács F. Nagy B. Sinkovics G. (1972). The gut bacterial flora of healthy early weaned piglets, with special regard to factors influencing its composition. Acta Vet. Acad. Sci. Hung. 22:327. 4667734

[B20] Ley R. E. Hamady M. Lozupone C. Turnbaugh P. J. Ramey R. R. Bircher J. S.. (2008). Evolution of mammals and their gut microbes. Science 320, 1647–1651. 10.1126/science.1155725 18497261 PMC2649005

[B21] Li C. W. Jiang Z. G. Fang J. M. Jiang G. H. Ding Y. H. Chen H. (2005b). Relationship between reproductive behavior and fecal steroid in milu (*Elaphurus davidianus*). Acta Theriol. 20, 88–100.

[B22] Li C. W. Jiang Z. G. Zeng Y. You Z. Q. (2005a). Rutting tactics in Père David's deer stags under different population densities and during different rut periods. Biodivers. Sci. 13, 424–431.

[B23] Li C. X. Wang W. H. Bai Z. J. Wang Z. Y. (2007). The entero toxemia of wild animal ruminant and its prevention and cure. Chin. J. Veterin. Med. 43:23.

[B24] Li K. Zhang S. M. Zhang D. D. Zhong Z. Y. (2007). Digestibility determination of *Elaphurus davidianus* for *Medicago sativa*. J. Econ. Anim. 11, 76–80.

[B25] Li Y. M. Hu X. L. Yang S. Zhou J. Zhang T. Qi L.. (2017). Comparative analysis of the gut microbiota composition between captive and wild forest musk deer. Front. Microbiol. 8:1705. 10.3389/fmicb.2017.01705 28928728 PMC5591822

[B26] Liu R. Duan J. A. Qian D. W. Peng Y. R. Ding Y. H. Shang E. X. (2011). Thoughts on resources and sustainable development of Chinese Père David's Deer. World Sci. Technol. 13, 213–220.

[B27] Malmuthuge N. Le L. G. (2017). Understanding host-microbial interactions in rumen: searching the best opportunity for microbiota manipulation. J. Anim. Sci. Biotechnol. 8, 300–306. 28116074 10.1186/s40104-016-0135-3 PMC5244612

[B28] Meng Y. P. Li K. Zhang L. Y. Yang M. Chen Q. (2010). Measurement and analysis on feed intake of *Elaphurus davidianus* in Beijing Milu park. Special Wild Econ. Anim. Plant Res. 4, 39–42.

[B29] Pitta D. W. Kumar S. Veiccharelli B. Parmar N. Reddy B. Joshi C. G.. (2014). Bacterial diversity associated with feeding dry forage at different dietary concentrations in the rumen contents of Mehshana buffalo (*Bubalus bubalis*) using 16S pyrotags. Anaerobe 25, 31–41. 10.1016/j.anaerobe.2013.11.008 24315806

[B30] Schloss P. D. Westcott S. L. Ryabin T. Hall J. R. Hartmann M. Hollister E. B.. (2009). Introducing Mothur: open-source, platform-independent, community-supported software for describing and comparing microbial communities. Appl. Environ. Microbiol. 75, 7537–7541. 10.1128/AEM.01541-09 19801464 PMC2786419

[B31] Sears C. L. (2005). A dynamic partnership: celebratingour gut flora. Anaerobe 11, 247–251. 10.1016/j.anaerobe.2005.05.001 16701579

[B32] Sundset M. A. Præsteng K. E. Cann I. K. Mathiesen S. D. Mackie R. I.. (2007). Novel rumen bacterial diversity in two geographically separated sub-species of reindeer. Microb. Ecol. 54, 424–438. 10.1007/s00248-007-9254-x 17473904

[B33] Turnbaugh P. J. Ley R. E. Mahowald M. A. Magrini V. Mardis E. R. Gordon J. I. (2006). Anobesity-associated gut microbiome with increased capacity for energy harvest. Nature 444, 1027–1131. 10.1038/nature05414 17183312

[B34] Wang L. B. Ding Y. H. Wei J. X. (2009). Restrictive factors on population development of muli deer in dafeng national nature reserve. Chin. J. Wildl. 30, 299–301.

[B35] Wang Y. Wang W. (2011). Diet of Pere David'S deer (*Elaphurus davidianus*) at Milu Park in Beijing, China. Chin. J. Wildl. 32, 65–68.

[B36] Wang Y. Z. Lu X. D. Duan X. (1991). Cases of Père David's deer hemorrhagic enteritis. Chin. J. Veter. Med. 5, 38–39.

[B37] Wei H. (2008). Studies of the Structural Changes of Gut Microbiota in Response to Various Perturbations. Shanghai: Shanghai Jiao Tong University.

[B38] Xu J. Bjursell M. K. Himrod J. Deng S. Carmichael L. K. Chiang H. C.. (2003). A genomic view of the human-bacteroides thetaiotaomicron symbiosis. Science 299, 2074–2076. 10.1126/science.1080029 12663928

[B39] Yang D. D. Ma J. Z. He Z. Li P. F. Wen H. J. Jiang Z. G. (2007). Population dynamics of the Père David's deer *Elaphurus davidianus* in Shishou Milu National Nature Reserve, Hubei Province, China. Acta Zool. Sin. 53, 947–952.

[B40] Yang M. F. Cui B. A. Wei Z. Y. Wang X. B. Zhang S. M. Xu D. H. (2004). Clostridium perfringens disease and pasteurellosis infection of Père David's deer. Chin. J. Veter. Med. 3, 51–52.

[B41] Zhang C. L. Zhao D. M. Li Q. Liu L. (1997). Analysis on the regularity of herbivorous animals in Beijing Zoo, in A Special Collection of Animal Pathology on the Law of Herbivorous Animals in Beijing Zoo, ed Zhang C. L. Zhao D. M. Li Q. Liu L. (Beijing: China Agricultural University press), 154–157.

[B42] Zhang L. Y. Zhang S. M. (2013). Research status of *ex-situ* conservation biology of Père David's Deer in China. China Sci. Technol. Achieve. 13, 8–10.

[B43] Zhang S. M. Bai J. D. Li Y. P. Chen Q. Cheng Z. B. Meng Q. H. (2012). Père David's Deer's *ex-situ* conservation status, conservation patterns and conservation suggestions in China. For. Resour. Manage. 39, 225–230.

[B44] Zheng Y. Zhang J. C. Guo Z. Zhang H. P. (2014). Research progress on high-throughput sequencing to analysis the diversity of gut microbiota and its influence factors. J. Chin. Insti. Food Sci. Technol. 14, 157–164.

[B45] Zhong Z. Y. Shan Y. F. Zhang L. Y. Cheng Z. B. Wang L. B. Zhang P. Q. (2015). Pathology observation of Père David's Deer's (*Elaphurus davidianus*) sudden death syndrome. *Periodical Press Shanxi Agricult. Univ*. (Natural Science Edition) 3, 290–296.

[B46] Zhong Z. Y. Zhang L. Y. Xia J. S. Tang B. T. Chen G. (2007). Epidemiological investigation of sudden death of Père David's deer in the South China Sea in 1999, in Père David's Deer Home 20th Anniversary International Academic Symposium eds Zhong Z. Y. Zhang L. Y. Xia J. S. Tang B. T. Chen G. (Beijing: Beijing Press), 34–37.

[B47] Zhou S. (2016). Effects and Correlative Research of Daidzein on Production Performance, Blood Index and Fecal Microfolar in Growing Jinjing Cattle. JiangXi: JiangXi Agricultural University.

